# Supplementary material for: Photothermally induced natural vibration for versatile and high-speed actuation of crystals
Source: Nat Commun. 2023 Mar 13;14:1354. doi: 10.1038/s41467-023-37086-8 (PMC10008822; doi:10.1038/s41467-023-37086-8)
Supplement: Supplementary file 1 — Supplementary Information [file 41467_2023_37086_MOESM1_ESM.pdf]

Supplementary Information

# Photothermally induced natural vibration for versatile and high-speed actuation of crystals

Yuki Hagiwara[1], Shodai Hasebe[1], Hiroki Fujisawa[2], Junko Morikawa[2], Toru Asahi[1,3], Hideko Koshima[3*]

[1] Graduate School of Advanced Science and Engineering, Waseda University, 3-4-1 Okubo, Shinjuku-ku, Tokyo 169–8555, Japan.

[2] School of Materials and Chemical Technology, Tokyo Institute of Technology, 2-12-1 Ookayama, Meguro-ku, Tokyo 152-8550, Japan.

[3] Research Organization for Nano & Life Innovation, Waseda University, 513 Wasedatsurumaki-cho, Shinjuku-ku, Tokyo 162-0041, Japan.

[*]Correspondence to Email: h.koshima@kurenai.waseda.jp

**Contents**

**1. Crystal structure**

Supplementary Table 1, Supplementary Fig. 1 ······ 3

**2. Measurements of thermal properties**

Supplementary Figs. 2–5, Supplementary Table 2 ······ 4

**3. Fluorescence spectrum measurement**

Supplementary Fig. 6 ······ 8

**4. Young's modulus measurement**

Supplementary Fig. 7, Supplementary Table 3 ······ 9

**5. Natural vibration induced by the photothermal effect**

Supplementary Figs. 8–12, Supplementary Tables 4–6 ······ 10

**6. Simulation of crystal bending**

Supplementary Figs. 13–15 ······ 17

**7. Supplementary references** ······ 24

# 1. Crystal structure

**Supplementary Table 1** Crystal structure of the **1β** crystal at 20 °C

| Temperature (°C) | 20 |
|---|---|
| Formula | $C_7H_6N_2O_5$ |
| Formula Weight | 198.14 |
| Crystal System | Monoclinic |
| Space Group | $P2_1/n$ |
| $a$ (Å) | 3.9723(9) |
| $b$ (Å) | 13.700(3) |
| $c$ (Å) | 15.431(4) |
| $\alpha$ (°) | 90 |
| $\beta$ (°) | 91.128(6) |
| $\gamma$ (°) | 90 |
| $Z$ | 4 |
| $V$ (Å$^3$) | 839.6(3) |
| $d_{calc}$ (g cm$^{-3}$) | 1.567 |
| $R_1$ [$I > 2\sigma$(I)] | 0.0604 |
| $wR_2$ [$I > 2\sigma$(I)] | 0.1400 |
| GOF | 1.038 |

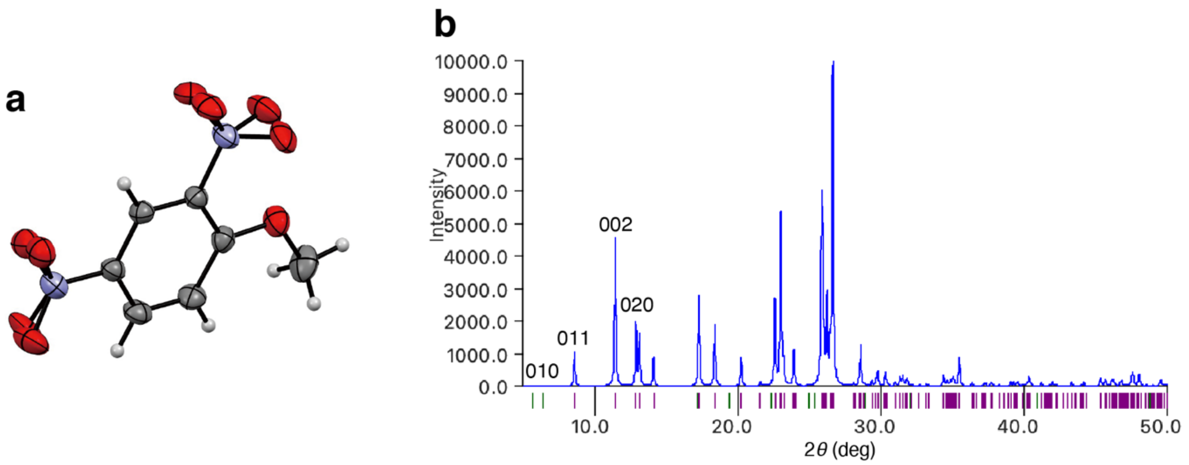


**Supplementary Fig. 1 a** ORTEP drawing of the molecule in the **1β** crystal at 20 °C with thermal ellipsoid of 25% probability. **b** PXRD pattern obtained from the X-ray crystallographic data.

## 2. Measurements of thermal properties

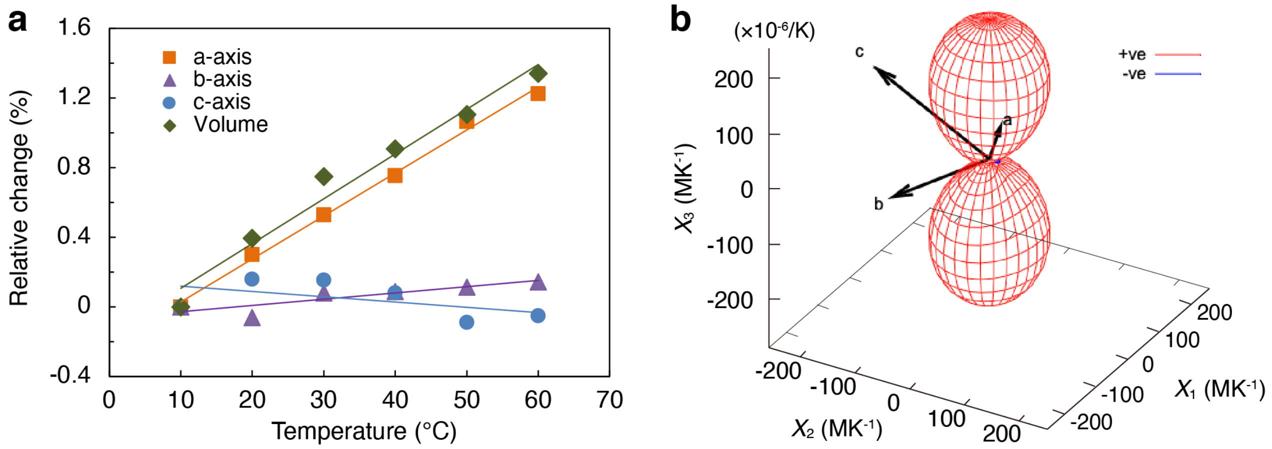


**Supplementary Fig. 2** Thermal expansion of **1β** crystal. **a** Relative change of three axes lengths and the volume by temperature change. **b** Thermal expansivity indicatrix calculated using PASCal.[S1]

**Supplementary Table 2** Summary table of temperature dependence of unit cell parameters and thermal expansion coefficients.

| Temperature (°C) | *a* (Å) | *b* (Å) | *c* (Å) | *V* (Å³) | *α* (°) | *β* (°) | *γ* (°) |
|---|---|---|---|---|---|---|---|
| 10 | 3.972(3) | 13.742(12) | 15.465(6) | 844(2) | 90.05(9) | 91.06(4) | 90.02(4) |
| 20 | 3.984(7) | 13.733(25) | 15.489(16) | 847(2) | 90.06(8) | 91.01(4) | 90.11(7) |
| 30 | 3.993(4) | 13.753(9) | 15.489(34) | 850(1) | 90.10(7) | 90.84(3) | 90.09(4) |
| 40 | 4.002(7) | 13.754(8) | 15.477(27) | 851(1) | 90.04(4) | 90.83(5) | 89.92(18) |
| 50 | 4.015(5) | 13.757(9) | 15.451(12) | 853(1) | 89.98(12) | 90.89(2) | 90.00(4) |
| 60 | 4.021(3) | 13.762(19) | 15.457(22) | 855(2) | 90.01(2) | 90.76(5) | 90.08(3) |
| Thermal expansion coefficient ($MK^{-1}$) | 247 | 36.0 | -30.5 | 257 | ----- | ----- | ----- |

( ): Standard deviation, *n* = 3

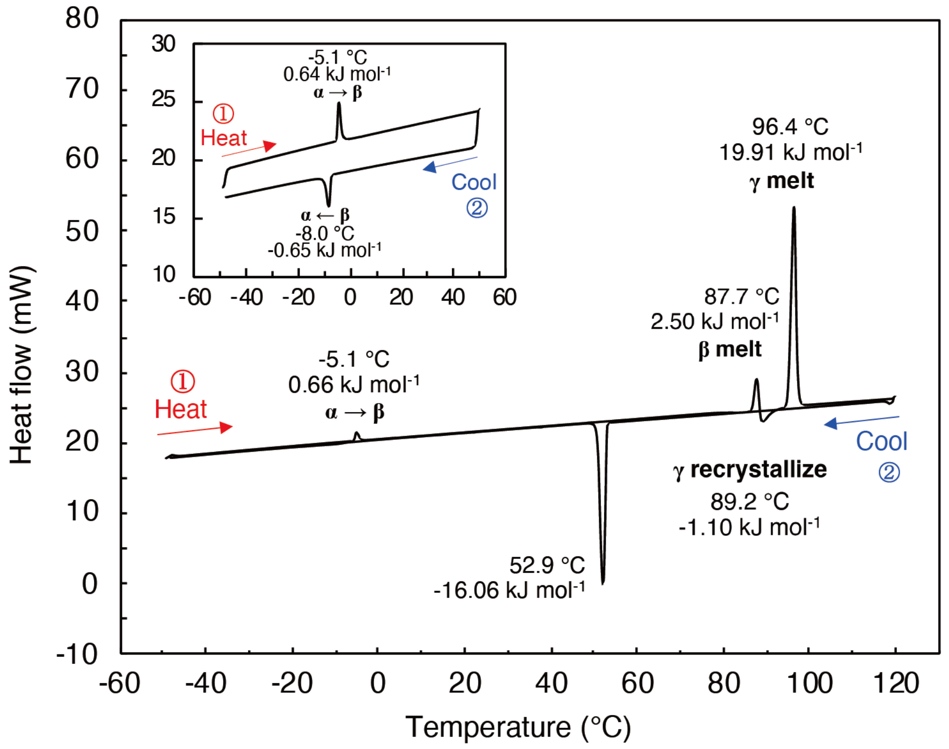


**Supplementary Fig. 3** Differential scanning calorimetry curve of crystals of **1** at a speed of 10 °C min$^{-1}$ on heating and subsequent cooling.

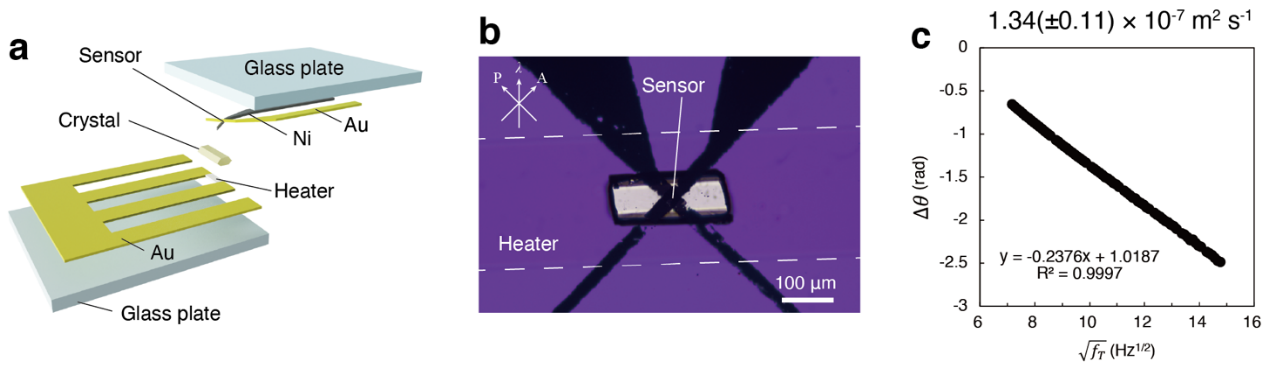


**Supplementary Fig. 4** Thermal diffusivity measurement of a **1β** crystal by temperature wave analysis (TWA).[S2–5] **a** Schematic assembly of samples for TWA measurement. **b** Polarized photo of a **1β** crystal (thickness: 49.2 μm) between the heater and the sensor. **c** The plot of phase delay $\Delta\theta$ and square root of temperature wave frequency $f_T$ in the range from 51.3 to 251.5 Hz ($kd$ = 1.71–3.94).

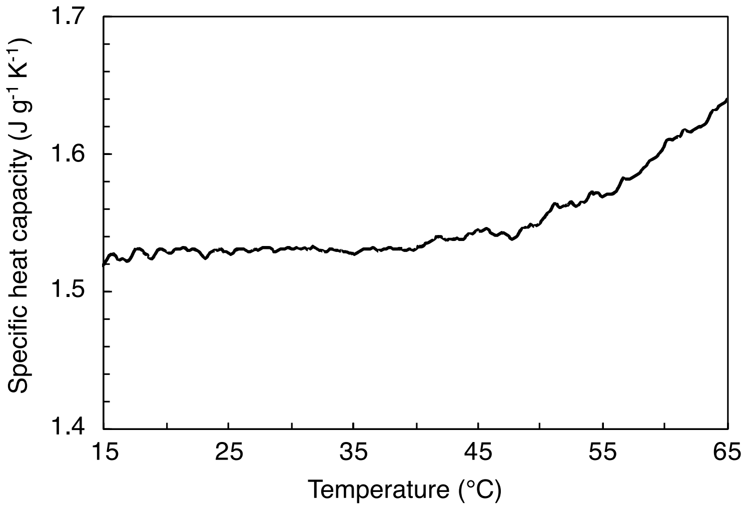


**Supplementary Fig. 5** Temperature dependence of heat capacity of **1β** crystals.

## 3. Fluorescence spectrum measurement

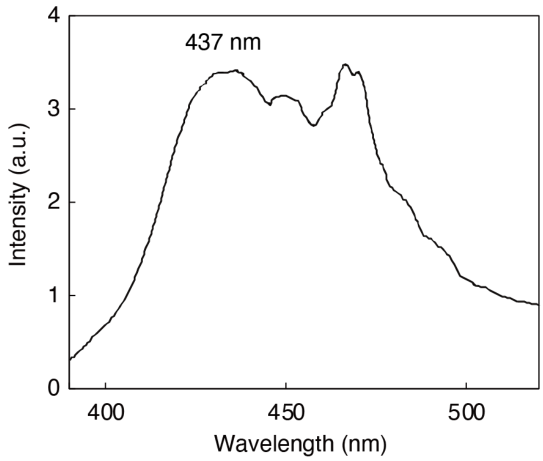


**Supplementary Fig. 6** Fluorescence spectrum ($\lambda_{ex}$ = 300 nm) of powdered **1β** crystals with a peak at 437 nm. Peaks around 450–480 nm are derived from the apparatus.

The fluorescence quantum yield ($\lambda_{ex}$ = 375 nm) of the **1β** single crystals was determined to be 0.005 with an absolute photoluminescence quantum yield spectrometer.

## 4. Young's modulus measurement

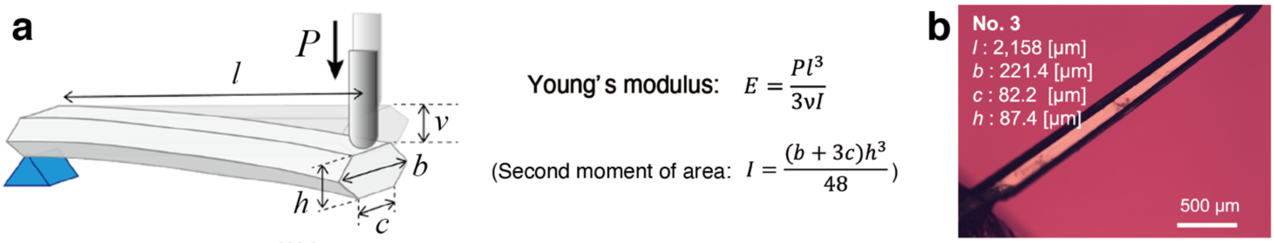


**Supplementary Fig. 7** Young's modulus measurement of **1β** crystals by cantilever bending test. **a** Illustration of the crystal cantilever with an applied force *P*. **b** Polarized photo of a measured crystal viewed from the top (sample No.3 in Supplementary Table 3).

**Supplementary Table 3** Summary table of the measured Young's modulus of crystals with various shapes.

| Sample No. | $n$ | Length $l$ (μm) | Width $b$ (μm) | Top width $c$ (μm) | Thickness $h$ (μm) | Young's modulus $E$ (GPa) | |
|---|---|---|---|---|---|---|---|
| | | | | | | Mean | SE |
| 1 | 6 | 3,497 | 258.8 | 134.9 | 193.2 | 1.68 | 0.06 |
| 2 | 5 | 2,650 | 177.3 | 67.2 | 130.2 | 1.69 | 0.10 |
| 3 | 6 | 2,158 | 221.4 | 82.2 | 87.4 | 1.71 | 0.14 |
| 4 | 6 | 1,835 | 181.1 | 57.2 | 83.7 | 1.48 | 0.04 |
| 5 | 4 | 2,130 | 126.4 | 31.0 | 90.7 | 1.71 | 0.10 |

Young's modulus of the **1β** crystal was measured using a force load machine (RTG-1210, A&D). The tip of a single crystal was fixed to a glass plate as a support, and the other tip was free to move vertically. The free tip was pressed in the vertical downward direction by the jig at the rate of 200 μm min$^{-1}$, and the jig displacement dependence of the load force was measured during the compression of the free tip. Such bending test used five crystals of **1β** and repeated several times for each crystal, and the Young's modulus was estimated to be **<u>1.65 (0.04) GPa</u>**.

## 5. Natural vibration induced by the photothermal effect

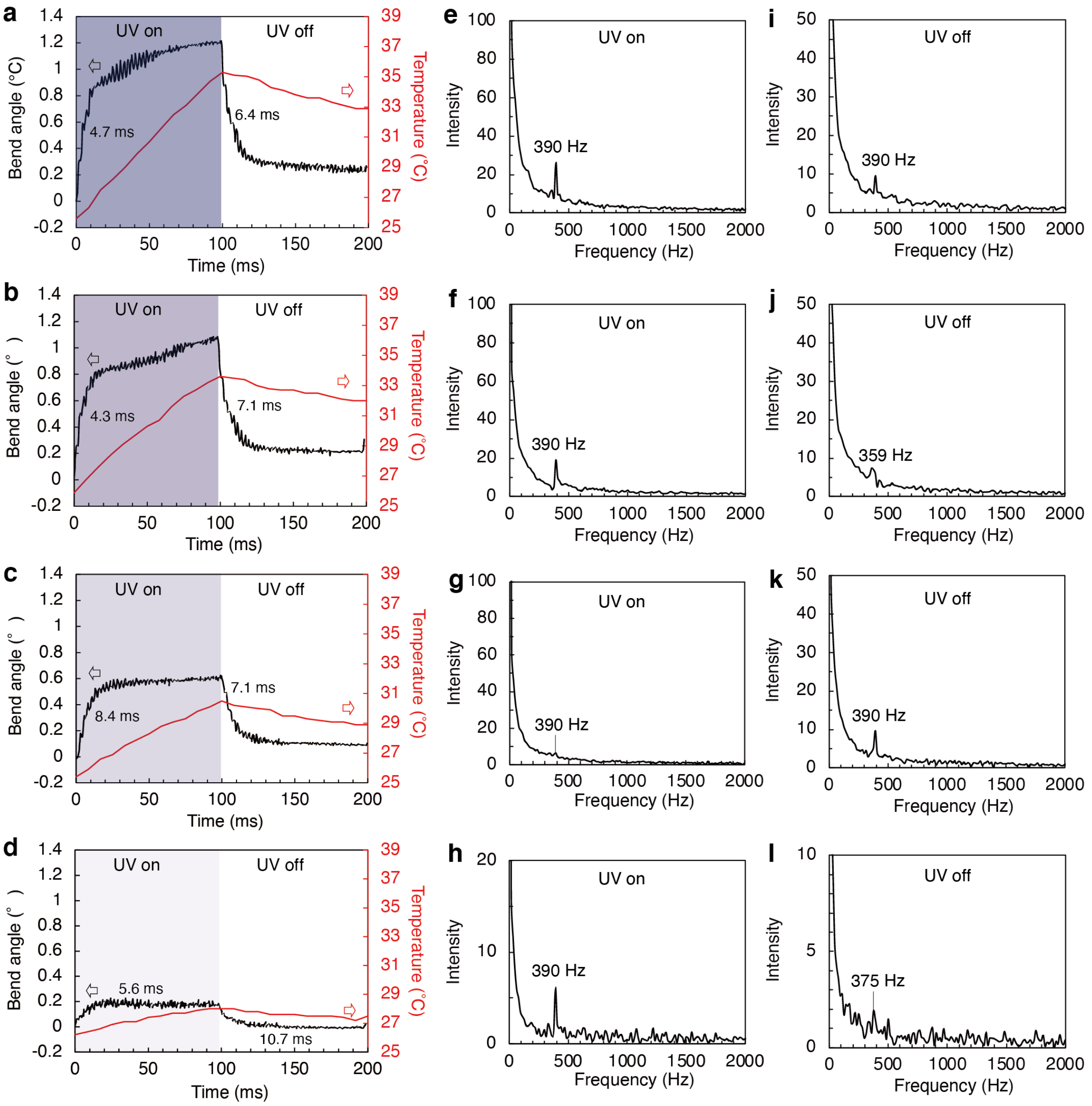

**Supplementary Fig. 8 a–d** Time profiles of crystal III bending (black line) and top surface temperature (red line) upon UV irradiation at (**a**) 1456, (**b**) 1219, (**c**) 642, (**d**) 160 mW cm$^{-2}$ for 100 ms and after the UV cessation. The numbers in the graphs indicate time constants. **e–l** Fourier transform analyses with and without UV irradiation at (**e, i**) 1456, (**f, j**) 1219, (**g, k**) 642, (**h, l**) 160 mW cm$^{-2}$ for 100 ms.

**Supplementary Table 4** Summary table of the photothermally driven bending with the natural vibration at various UV intensities.

| UV intensity (mW cm$^{-2}$) | Large photothermal bending | | Natural vibration UV on | | Natural vibration UV off | |
|---|---|---|---|---|---|---|
| | Maximum bend angle (°) | maximum temperature (°C) | Maximum bend angle (°) | Natural frequency (Hz) | Maximum bend angle (°) | Natural frequency (Hz) |
| 1456 | 1.218 | 35.3 | 0.198 | 390 | 0.173 | 390 |
| 1219 | 1.085 | 33.6 | 0.152 | 390 | 0.119 | 359 |
| 642 | 0.624 | 30.5 | 0.101 | 390 | 0.0770 | 390 |
| 160 | 0.226 | 27.5 | 0.0595 | 390 | 0.0410 | 375 |

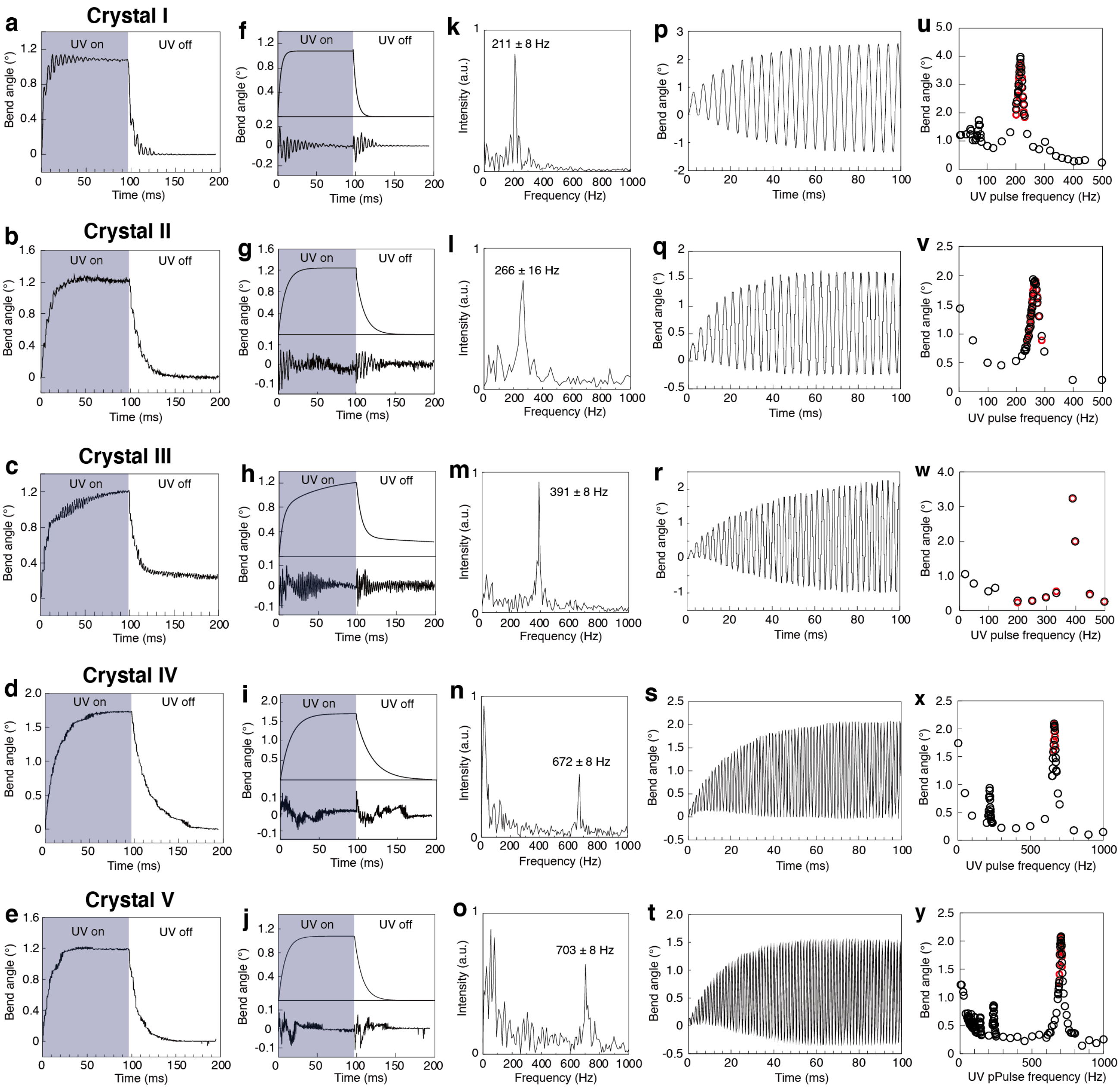


**Supplementary Fig. 9** Photothermally driven bending of various shape crystals I–V with natural vibrations of **I.** 216, **II**. 268, **III**. 390, **IV.** 664, **V.** 702 Hz. The detail shape information is shown in Supplementary Table 2. (**a–e**) Time dependence of the bending angle under and after irradiation of UV for 100 ms. (**f–j**) Time profile of the fitted exponential curve of the large photothermally driven bending (upper) and the extracted natural vibration (lower) of **a–e**. (**k–o**) Graphs of Fourier transform analyses of **a–e**. (**p–t**) Time dependence of the resonated bending of the natural vibration under pulsed UV irradiation of the natural frequency. (**u–y**) Relationship between pulse frequency and maximum bending angle: experimental (black) and fitted (red) bending angles (see the footnote in Supplementary Table 2).

**Supplementary Table 5** Summary of crystal shape, photothermally driven bending, non-resonated natural vibration, and resonated natural vibration of crystals I–V.

| | I | II | III | IV | V |
|---|---|---|---|---|---|
| Crystal shape | | | | | |
| Length $l$ /μm | 5,100 | 8,419 | 6,075 | 8,180 | 5,444 |
| Width $b$ /μm | 163 | 178 | 151 | 295 | 298 |
| Top width $c$ /μm | 47 | 116 | 40 | 127 | 173 |
| Thickness $h$ /μm | 48 | 124 | 105 | 215 | 136 |
| Weight $m$ /μg | 40.3 | 240 | 95.5 | 581 | 273 |
| Photothermally driven bending | | | | | |
| Bend angle under UV for 100ms /° | 1.145 | 1.298 | 1.216 | 1.741 | 1.224 |
| Time constants $\tau_{on}$ /ms | 3.5 | 8.7 | 4.7 | 13.2 | 8.5 |
| Time constants $\tau_{off}$ /ms | 4.1 | 10.7 | 6.4 | 19.4 | 9.9 |
| Tip speed $v_l$ /m s$^{-1}$ | 0.029 | 0.022 | 0.013 | 0.019 | 0.014 |
| Energy conversion efficiency $\eta$ | $1.56 \times 10^{-7}$ | $4.79 \times 10^{-7}$ | $1.14 \times 10^{-7}$ | $1.28 \times 10^{-6}$ | $3.90 \times 10^{-7}$ |
| Non-resonated natural vibration | | | | | |
| Measured natural frequency $f$ /Hz | 216 | 266 | 390 | 664 | 702 |
| Fitted natural frequency $f_{fit}$ /Hz **a)** | 216 | 268 | 390 | 666 | 702 |
| Calculated natural frequency $f_{cal}$ /Hz | 262 | 274 | 397 | 476 | 707 |
| Bend angle under UV for 100ms /° | 0.316 | 0.201 | 0.198 | 0.094 | 0.065 |
| Tip speed $v_l$ /m s$^{-1}$ | 0.019 | 0.025 | 0.026 | 0.028 | 0.014 |
| Energy conversion efficiency $\eta$ | $6.73 \times 10^{-8}$ | $6.16 \times 10^{-7}$ | $4.66 \times 10^{-7}$ | $2.85 \times 10^{-6}$ | $3.86 \times 10^{-7}$ |
| Resonated natural vibration | | | | | |
| Bend angle under UV pulse of $f$ Hz /° | 3.984 | 1.851 | 3.381 | 2.054 | 2.082 |
| Resonance amplification ratio | 12.6 | 9.21 | 17.1 | 21.9 | 32 |
| Tip speed $v_l$ /m s$^{-1}$ | 0.24 | 0.23 | 0.41 | 0.62 | 0.44 |
| Energy conversion efficiency $\eta$ | $1.05 \times 10^{-5}$ | $5.22 \times 10^{-5}$ | $1.19 \times 10^{-4}$ | $1.37 \times 10^{-3}$ | $4.05 \times 10^{-4}$ |

**a)** To calculate the fitted natural frequency $f_{fit}$, the relationship between UV pulse frequency and the measured maximum bending angle (Supplementary Fig. 9u–y) was fitted to the model of the forced vibration with the damping, as shown in the equation below:

$$\theta(f_p) = \frac{\theta_{st}}{\sqrt{\left\{1-\left(\frac{f_p}{f_{fit}}\right)^2\right\}^2 + \left\{2\zeta\left(\frac{f_p}{f_{fit}}\right)^2\right\}^2}}$$

where $f_p$ is the UV pulse frequency, $\theta(f_p)$ is the maximum bending angle at the certain pulse frequency, and $\theta_{st}$ and $\zeta$ are fitting parameters: the static deflection angle and the damping ratio, respectively. As a result, the fitted natural frequency is firmly coincident with the measured natural frequency.

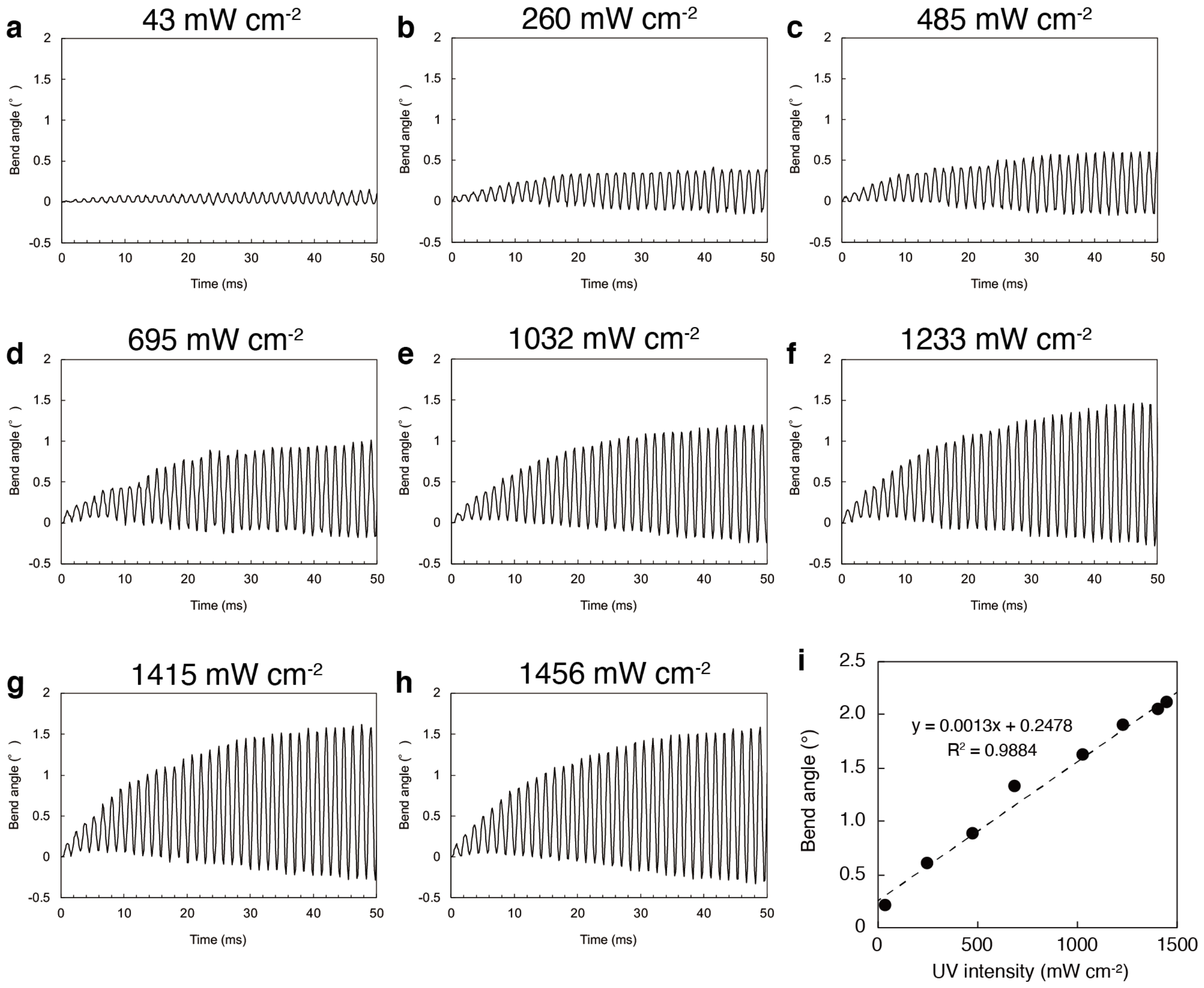


**Supplementary Fig. 10 a–h** Time profiles of resonated natural vibration of crystal V upon 702 Hz pulsed UV irradiation at (**a**) 43, (**b**) 260, (**c**) 485, (**d**) 695, (**e**) 1032, (**f**) 1233, (**g**) 1415, (**h**) 1456 mW cm$^{-2}$. **i** UV light intensity dependence of the maximum bending angle upon 702 Hz pulse UV irradiation.

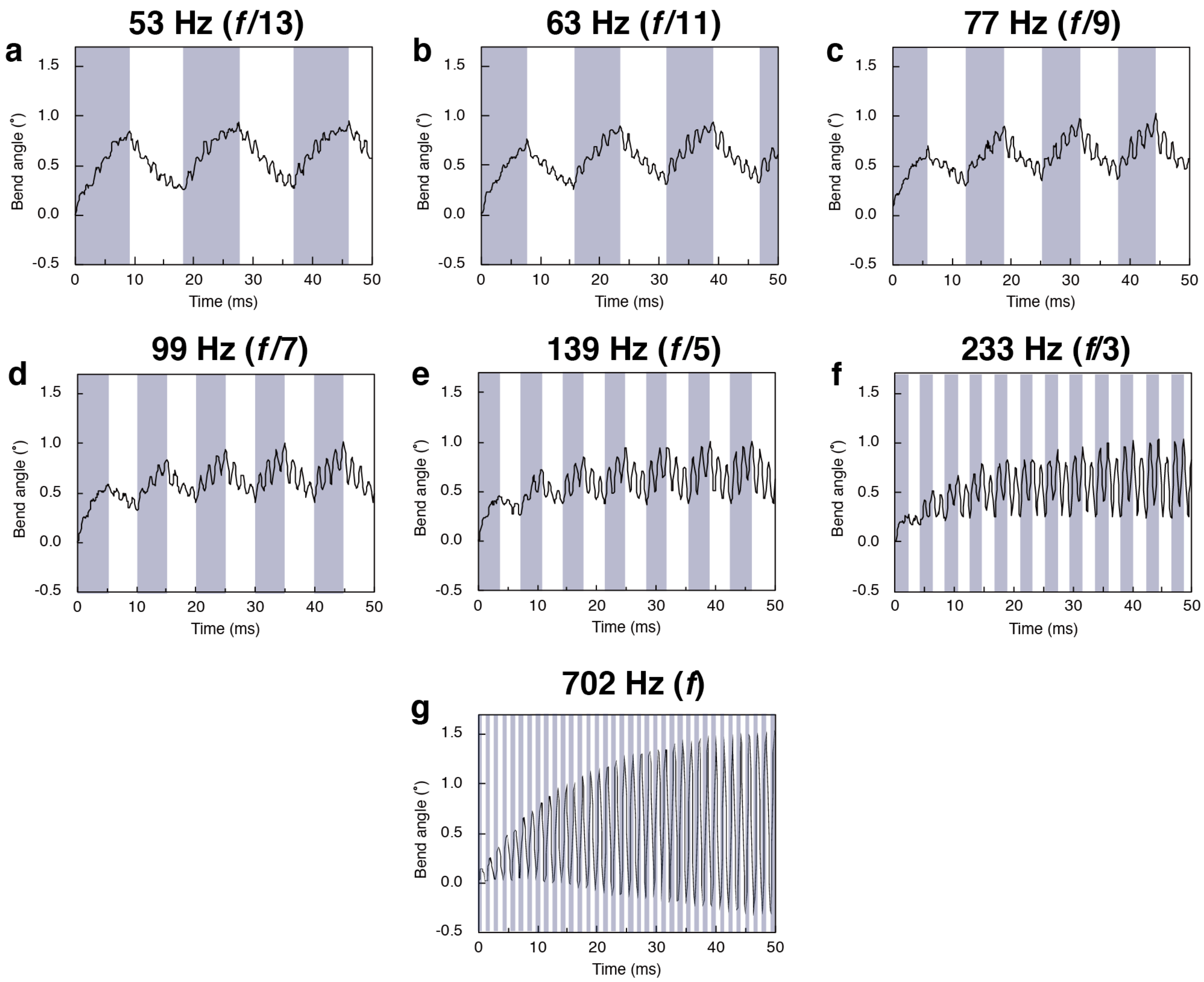


**Supplementary Fig. 11** Time profiles of high-speed bending of crystal V upon UV pulse irradiation of (**a**) 53, (**b**) 63, (**c**) 77, (**d**) 99, (**e**) 139, (**f**) 233 Hz, (**g**) 702 Hz.

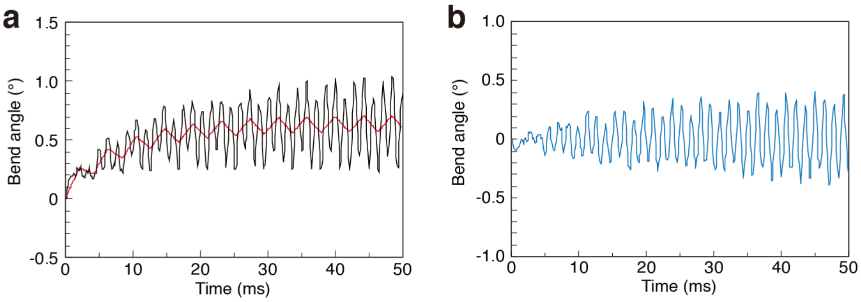


**Supplementary Fig. 12** Evaluation method of magnification of the natural vibration during high-speed bending of odd fractions of the natural frequency (e. g. 233 Hz of crystal V). **a** Experimental high-speed bending (black) and the fitted photothermally driven bending (red) under pulsed UV irradiation. **b** Time profile of the extracted natural vibration.

Based on the results of the exponential fitting of the bending profile when irradiated with UV light for 100 ms, we constructed a fitting system for producing the fitted photothermal bending under any frequency UV pulse. According to the fitted bending angle under UV irradiation for 100 ms, the maximum bending angle $A$ and time constants $\tau_{on}$ and $\tau_{off}$ were provided as constants. The bending angle $\theta_{on}$ ($t_{on}$) under UV irradiation and the bending angle $\theta_{off}$ ($t_{off}$) after UV irradiation were represented, as shown in equations (5.1) and (5.2), respectively:

$$\theta_{on}(t_{on}) = A\left(1 - \exp\left(-\frac{t}{\tau_{on}}\right)\right) \tag{5.1}$$

$$\theta_{off}(t_{off}) = A\exp\left(-\frac{t}{\tau_{off}}\right) \tag{5.2}$$

For reproducing the photothermal bending under UV pulse irradiation, the time $t_{on}$ and $t_{off}$ was corrected to substitute corrected times to equations (5.1) and (5.2) every time UV on/off is switched, using equations (5.3) and (5.4):

$$t_{on} = -\tau_{on}\ln\left(1 - \frac{\theta_{on}(t_{on})}{A}\right) \tag{5.3}$$

$$t_{off} = -\tau_{off}\ln\frac{T - T_0}{A} \tag{5.4}$$

As a result, the fitted photothermal bending was reproduced to behave periodically accompanying with the gradual bending angle increase, as shown in red curve in Supplementary Fig.12a. The bending angle of natural vibration was extracted by subtracting the bending angle of the fitted photothermal bending from the experimental bending angle (black, Supplementary Fig. 12a), as shown in Supplementary Fig. 12b.

**Supplementary Table 6** Summary of the tip deflection speed, energy conversion efficiency and crystal shape of **1β** and other previously reported crystals.[S5–7]

| Crystal | | Tip speed /m $s^{-1}$ | Energy conversion efficiency | Shape | | |
|---|---|---|---|---|---|---|
| | | | | Length /μm | Width /μm | Thickness / μm |
| Resonated natural vibration | | | | | | |
| **1β** | I | 0.24 | $1.05 \times 10^{-5}$ | 5,100 | 163 | 48 |
| | II | 0.23 | $5.22 \times 10^{-5}$ | 8,419 | 178 | 124 |
| | III | 0.41 | $1.19 \times 10^{-4}$ | 6,075 | 151 | 105 |
| | IV | 0.62 | $1.37 \times 10^{-3}$ | 8,180 | 295 | 215 |
| | V | 0.44 | $4.05 \times 10^{-4}$ | 5,444 | 298 | 136 |
| Non-resonated natural vibration | | | | | | |
| **1β** | I | 0.019 | $6.73 \times 10^{-8}$ | 5,100 | 163 | 48 |
| | II | 0.025 | $6.16 \times 10^{-7}$ | 8,419 | 178 | 124 |
| | III | 0.026 | $4.66 \times 10^{-7}$ | 6,075 | 151 | 105 |
| | IV | 0.028 | $2.85 \times 10^{-6}$ | 8,180 | 295 | 215 |
| | V | 0.014 | $3.86 \times 10^{-7}$ | 5,444 | 298 | 136 |
| Photothermally driven bending | | | | | | |
| **1β** | I | 0.029 | $1.57 \times 10^{-7}$ | 5,100 | 163 | 48 |
| | II | 0.022 | $4.79 \times 10^{-7}$ | 8,419 | 178 | 124 |
| | III | 0.013 | $1.14 \times 10^{-7}$ | 6,075 | 151 | 105 |
| | IV | 0.019 | $1.28 \times 10^{-6}$ | 8,180 | 295 | 215 |
| | V | 0.014 | $3.90 \times 10^{-7}$ | 5,444 | 298 | 136 |
| **2** | | 0.013 | $1.05 \times 10^{-7}$ | 1,912 | 48.2 | 35.4 |
| **3β** | | 0.046 | $1.04 \times 10^{-6}$ | 5,600 | 65 | 34 |
| **4** | | 0.003 | $4.98 \times 10^{-7}$ | 2,910 | 331 | 194 |
| **5** | | 0.001 | $1.85 \times 10^{-7}$ | 2,030 | 344 | 174 |
| Photoisomerization | | | | | | |
| **2** | | $6.4 \times 10^{-4}$ | $1.72 \times 10^{-12}$ | 1,170 | 14.5 | 10.2 |
| **3α** | | $1.4 \times 10^{-4}$ | $7.24 \times 10^{-16}$ | 167 | 15 | 0.5 |
| **4** | | $8.9 \times 10^{-4}$ | $5.90 \times 10^{-11}$ | 385 | 30 | 3.8 |
| **5** | | $2.5 \times 10^{-4}$ | $4.19 \times 10^{-12}$ | 171 | 17.2 | 2.4 |

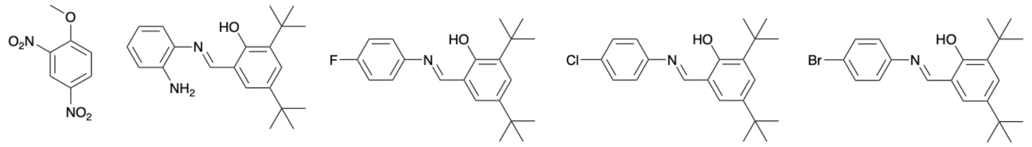


**1** **2** [S5] **3** [S6] **4** [S7] **5** [S7]

## 6. Simulation of crystal bending

The finite element analysis (FEA) is a method of discretizing an object by dividing it into multiple meshes, creating equations of balance for each mesh, and then solving the equations for the entire structure assembled from these meshes. In this research, the bending simulation is performed based on FEA using ANSYS analysis software.[S8]

### Ⅰ. Preparation of the crystal cantilever sample

To conduct FEA, physical properties of **1β** crystals and the three-dimensional geometry are required. The thermal diffusivity is applied for the non-steady heat conduction analysis, and density, thermal expansion coefficient, and Young's modulus are applied for the coupled thermo-structural analysis. The three-dimensional geometry of the hexagonal cantilever is prepared by the computer aided design software SpaceClaim.[S8]

### Ⅱ. Temperature gradient estimation by the non-steady heat conduction analysis

To perform the non-steady heat conduction analysis, suitable meshing is required for heat conduction along the thickness direction. In this research, the cross-section surface was equally meshed into 4 μm square (Supplementary Fig. 13a). The initial temperature $T_0$ was set to be the temperature of the crystal area in an IR thermography before UV laser irradiation, 25.6 °C. The atmospheric temperature was set to be the constant temperature of the atmospheric area, 25.2 °C. For temperature gradient estimation, the fitted temperatures obtained by results of thermography were used to adopt temperatures of the top and two upper slanting surfaces.

### Ⅲ. Bending angle calculation by the coupled thermo-structural analysis

The crystal cantilever deformation is calculated by the coupled thermo-structural analysis, the time history response analysis using thermal load as an external force estimated from the result of the previous non-steady heat conduction analysis. This analysis evaluates time dependence of shape deformation of the meshed crystal cantilever. Meshing and boundary condition are described as Supplementary Fig. 13b. At first, the long direction of the crystal cantilever was equally meshed by 50 μm per mesh. The cross-section surface was also meshed to several meshes to reflect the temperature gradient along the thickness direction. Then, one tip of the cross-section surface is set to be the fixed tip, and the result of the non-steady heat conduction analysis is applied as the ramped thermal load. The gravitational acceleration was applied in the vertical downward direction for the precise consideration of the tip displacement. The damping ratio $\zeta$ is set as an attenuation factor in reference to the equation (6.1):

$$\zeta = \frac{\gamma}{4\pi f} \tag{6.1}$$

where $\gamma$ is the Rayleigh damping (fitting parameter), and $f$ is the natural frequency.

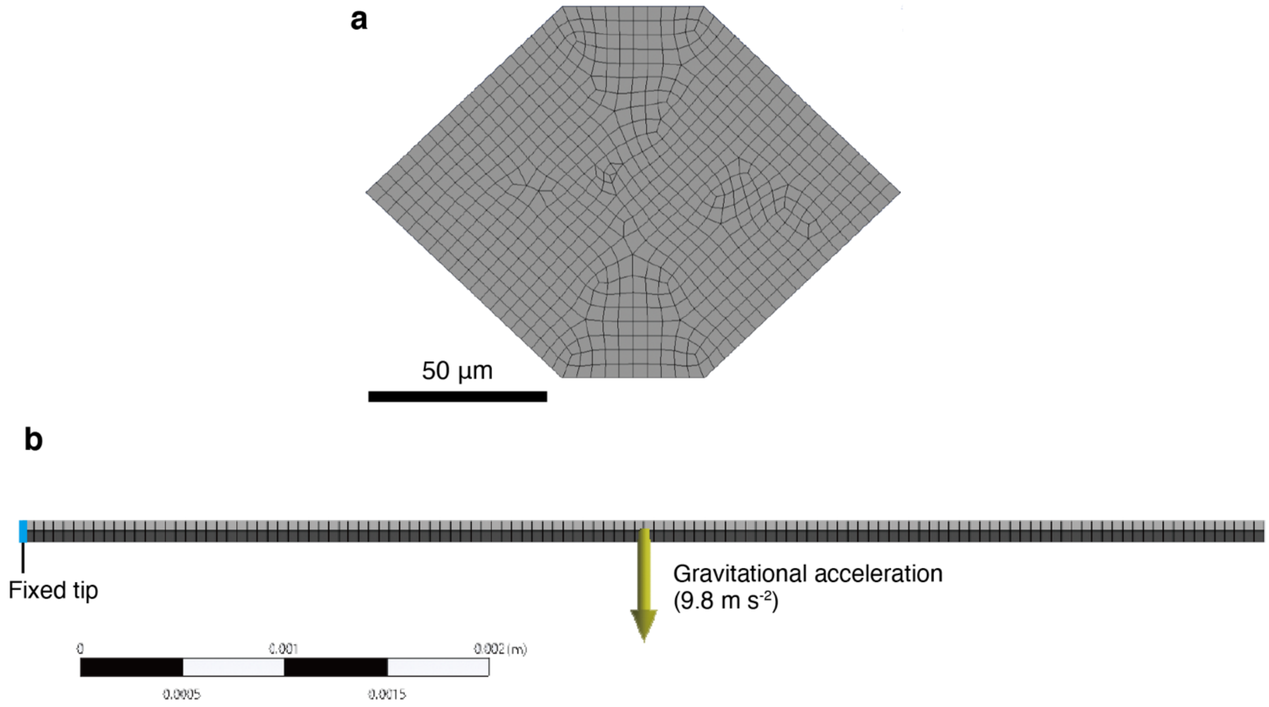


**Supplementary Fig. 13** Meshing and boundary condition for FEA. **a** Meshing of the cross-section surface for the non-steady heat conduction analysis. **b** Meshing along the length direction and boundary conditions for the coupled thermo-structural analysis.

## 6.1. Simulation I of bending of crystal III based on surface temperature measurement

For the simulation I, three types of input temperatures were determined from the measured temperature distribution by an IR thermography camera for setting to three sections of the top and two upper slanting surfaces (A, B, and C) (Supplementary Fig. 14a). First, the temperature distribution along the width direction was considered from results of the IR thermography (Supplementary Fig. 14b). The position dependence of the temperature was extracted on the measured line (10 points) upon UV irradiation for 100 ms (Supplementary Fig. 14c) and fitted as the trapezoid (two linear regression lines and three constant lines), resulting in determination of the crystal position and the temperature set points of the areas A, B, and C ($T_A$, $T_B$, and $T_C$, respectively) (Supplementary Fig. 14d). Thus, $T_A$ is set to the same temperature as the top surface temperature due to the trapezoid approximation. However, $T_B$ and $T_C$ are not placed on the measurement points I–VI and could not directly adopt the temperatures at measured points. For determining $T_B$ and $T_C$, time dependence of temperatures at the six points I–VI (Supplementary Fig. 14e) were fitted to exponential curves for extracting position dependence of the fitted values of $A$, $\tau_{UV\,on}$, and $\tau_{UV\,off}$, in accordance with the fitted single-exponential equations (6.2) and (6.3), the temperature change upon UV irradiation ($T_{UV\,on}$) and after the cessation of UV light ($T_{UV\,off}$), respectively:

$$T_{UV\,on}(t) = A\left(1 - \exp\left(-\frac{t}{\tau_{UV\,on}}\right)\right) + T_0 \tag{6.2}$$

$$T_{UV\,off}(t) = (T_{max} - T_0)\exp\left(-\frac{t}{\tau_{UV\,off}}\right) + T_0 \tag{6.3}$$

where $t$ is the time, $T_{UV\,on}(t)$ is the temperature upon UV irradiation at a certain $t$, $A$ is the constant value, $\tau_{UV\,on}$ is the time constant of the temperature increase upon UV irradiation, and $T_0$ is the initial temperature of the crystal (25.6 °C), $T_{UV\,off}(t)$ is the temperature after removing UV irradiation at a certain $t$, $T_{max}$ is the temperature upon UV irradiation for 100 ms, coincident with the maximum temperature of each point in Supplementary Fig. 14d, $\tau_{UV\,off}$ is the time constant of the temperature decrease after removing UV irradiation. Supplementary Fig. 14f, g show the position dependence of the fitted values $A$ and $\tau_{UV\,on}$, respectively, and both graphs were fitted to quadratic equations (inset functions in Supplementary Fig. 14f, g) at high $R^2$ values. As a result, temperatures of areas B and C upon UV irradiation ($T_{B,\,UV\,on}$ and $T_{C,\,UV\,on}$, respectively) are described as equations (6.4) and (6.5), respectively:

$$T_{B,UV\,on}(t) = 26.1\left(1 - \exp\left(-\frac{t}{239\text{ ms}}\right)\right) + 25.6\ [°\text{C}] \tag{6.4}$$

$$T_{C,UV\,on}(t) = 17.5\left(1 - \exp\left(-\frac{t}{181\text{ ms}}\right)\right) + 25.6\ [°\text{C}] \tag{6.5}$$

Supplementary Fig. 14h indicates the relationship between $T_{max}$-$T_0$ and $\tau_{UV\,off}$ and was highly fitted to linear regression (inset function). As a result, temperatures of areas B and C after stopping UV irradiation ($T_{B,\,UV\,off}$ and $T_{C,\,UV\,off}$, respectively) are described as equations (6.6) and (6.7), respectively:

$$T_{B,UV\,off}(t) = 8.93\exp\left(-\frac{t}{373\text{ ms}}\right) + 25.6\ [°\text{C}] \tag{6.6}$$

$$T_{C,UV\,off}(t) = 7.44\exp\left(-\frac{t}{315\text{ ms}}\right) + 25.6\ [°\text{C}] \tag{6.7}$$

In summary, the time profiles of temperatures on three areas (A, B, and C) are shown in Supplementary Fig. 14i.

For estimating temperature $T$ under UV pulse irradiation, time to substitute in equations (6.2) and (6.3) was returned according to equations (6.8) and (6.9), respectively, every time UV on/off is switched (at each half time of period: 1.282 ms):

$$t_{UV\,on} = -\tau_{UV\,on} \ln\left(1 - \frac{T - T_0}{A}\right) \tag{6.8}$$

$$t_{UV\,off} = -\tau_{UV\,off} \ln\frac{T - T_0}{T_{max} - T_0} \tag{6.9}$$

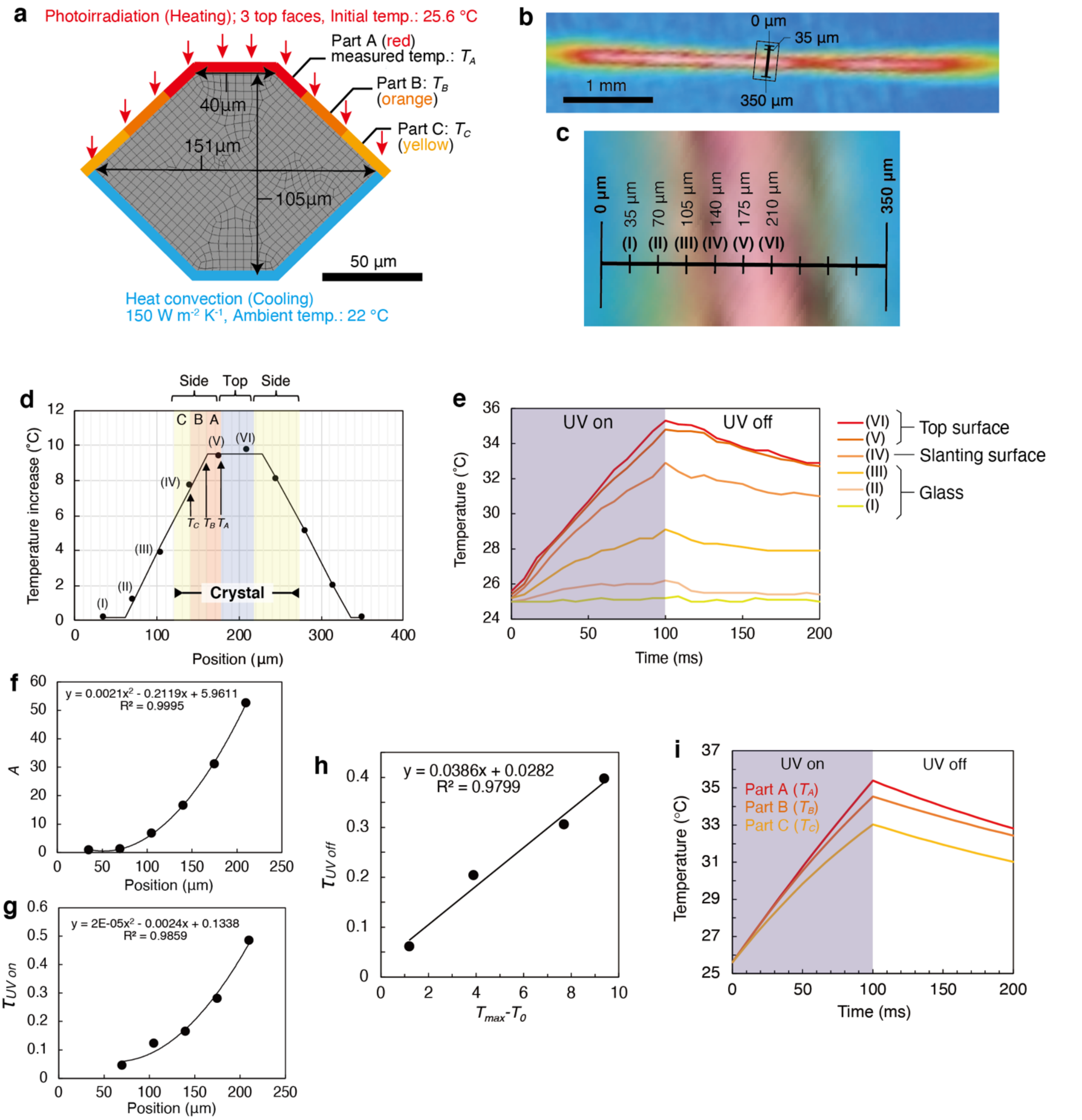


**Supplementary Fig. 14** Relationship between position along the width direction and temperature change. **a** Geometry of the cross-section surface with temperature input areas of A, B, and C. Arrows of $T_A$, $T_B$, and $T_C$ show temperature determination points of areas A, B, and C, respectively. **b** Temperature distribution of the crystal and the ambient air upon UV irradiation for 100 ms. Black line is coincident with the temperature measurement line along the width direction. Inset numbers indicate the position in accordance with the horizontal axis of **d.** **c** Enlarged view of the square in **b**. **d** Position dependence of the temperature increase upon UV irradiation for 100 ms. Blue region indicates the top face (width: 40 µm), and the red, orange, and yellow regions are the side area of A, B, and C, respectively. Arrows of $T_A$, $T_B$, and $T_C$ indicate temperature determination points of areas A, B, and C, respectively. Inset symbols indicate the temperature measurement points which correspond to the legend of **c**. **e** Time dependence of temperatures of six points I–VI. **f, g** Position dependence of the maximum temperature increase $A$ (**f**) and the time constant $\tau_{UV\,on}$ (**g**) upon UV irradiation. **h** Position dependence of the time constant $\tau_{UV\,off}$ after stopping UV irradiation. **i** Time dependence of temperature changes on part A, B, and C ($T_A$, $T_B$, and $T_C$, respectively).

## 6.2. Simulation II of bending of crystal III based on the irradiated light energy

For the simulation II, the photothermally generated heat was considered to be coincident with the irradiated light intensity, and the heat flux was applied to the top and two upper slanting faces as the photothermally generated heat, summarized in Supplementary Fig. 15a. The UV light was irradiated straight to the top surface of the crystal, so the heat flux to the top face was set to be the same value as the irradiated light intensity (14,560 W $m^{-2}$). Unlike the top face, UV light irradiated to two upper slanting surface from the direction of the incident angle (43.67°), thus the heat flux to those surfaces was set to be (14,560 W $m^{-2}$ × cos 43.67° =) 10,532 W $m^{-2}$. The heat convection was considered to be occurred on three bottom surfaces for fitting.

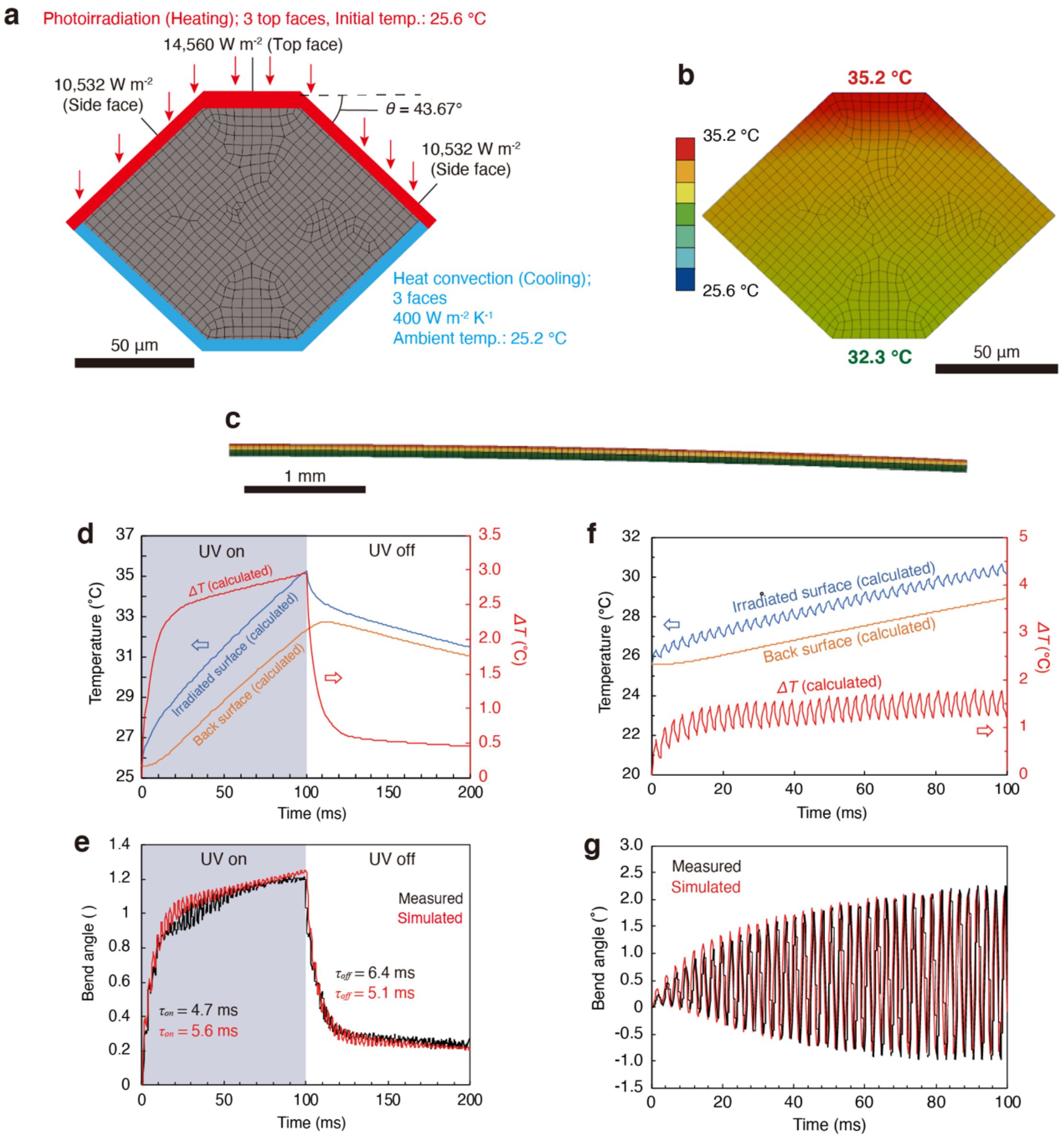


**Supplementary Fig. 15** Simulation II of **1β** crystal III bending by the photothermal effect and the natural vibration, based on the irradiation light intensity. **a** Meshing and heat condition for non-steady heat conduction analysis on simulation Ⅱ. **b, c** Simulated temperature distribution after UV laser irradiation for 100 ms: the (100) cross-section (**b**) and the side view of bent crystal (**b**). **d, e** Bending simulation with and without UV irradiation for 100 ms: time dependence of top surface (blue, measured), back surface (orange, calculated) temperature and temperature difference between two surfaces (*ΔT*, red, calculated) (**d**), time profiles of measured (black) and simulated (red). The inset values of $\tau_{on}$ and $\tau_{off}$ indicate the time constants for bending and straightening (**e**). **f, g** Simulation of amplified bending under 390 Hz pulse UV irradiation: time dependence of top surface (blue, measured), back surface (orange, calculated) temperature, and the temperature difference between two surfaces (*ΔT*, red) (**f**), time profiles of measured (black) and simulated (red), amplified bending angles (**g**).

## 7. Supplementary references


S1. Cliffe, M. J. & Goodwin, A. L. *PASCal*: a principal axis strain calculator for thermal expansion and compressibility determination, *J. Appl. Cryst.* **45**, 1321–1329 (2012).

S2. Morikawa, J., Hashimoto, T., Kishi, A., Shinoda, Y., Ema, K. & Takezoe, H. Critical anomalies in thermal diffusivity of liquid- crystalline terephthal-bis-(4-n-butylaniline). *Phys. Rev. E: Stat., Nonlinear, Soft Matter Phys.* **87**, 022501 (2013).

S3. Ryu, M., Batsale, J. C. & Morikawa, J. Quadrupole modelling of dual lock-in method for the simultaneous measurements of thermal diffusivity and thermal effusivity. *Int. J. Heat Mass Transfer* **162**, 120337 (2020).

S4. Ryu, M., Takamizawa, S. & Morikawa, J. Thermal diffusivity of organosuperelastic soft crystals during stress-induced phase transition. *Appl. Phys. Lett.* **119**, 251902 (2021).

S5. Hasebe, S., Hagiwara, Y., Komiya, J., Ryu, M., Fujisawa, H., Morikawa, J., Katayama. T., Yamanaka, D., Furube, A., Sato, H., Asahi, T. & Koshima, H. Photothermally Driven High-Speed Crystal Actuation and Its Simulation, *J. Am. Chem. Soc.* **143**, 8866–8877 (2021).

S6. Hasebe, S., Hagiwara, Y., Takechi, K., Katayama, T., Furube, A., Asahi, T. & Koshima, H. Polymorph-Derived Diversification of Crystal Actuation by Photoisomerization and the Photothermal Effect. *Chem. Mater.* **34**, 1315–1324 (2022).

S7. Hasebe, S., Hagiwara, Y., Hirata, K., Asahi, T. & Koshima, H. Crystal Actuation Switching by Crystal Thickness and Light Wavelength. *Mater. Adv.* **3**, 7098–7106 (2022).

S8. ANSYS® Academic Research Mechanical, 2021 R1 (ANSYS Inc., Cannonsburg, USA, PA, 2021).
