# Supplementary material for: Photothermally induced natural vibration for versatile and high-speed actuation of crystals
Source: Nat Commun. 2023 Mar 13;14:1354. doi: 10.1038/s41467-023-37086-8 (PMC10008822; doi:10.1038/s41467-023-37086-8)
Supplement: Supplementary file 3 — Description of Additional Supplementary Files [file 41467_2023_37086_MOESM3_ESM.pdf]

Description of Additional Supplementary Files

File Name: Supplementary Data 1
Description: Crystal structure of 1β at 20 °C.

File Name: Supplementary Movie 1
Description: Non-resonated natural vibration associated with large photothermally driven bending of 1β crystal III upon UV laser (375 nm, 1456 mW cm-2) irradiation for 100 ms and then turning off the UV light, and simultaneous monitor of surface temperature with an IR thermography camera (realtime → slow motion × 0.01) (MP4).

File Name: Supplementary Movie 2
Description: Resonated natural vibration of 1β crystal III upon 390 Hz pulsed (1.282 ms on, 1.282 ms off) UV laser (375 nm, 1,456 mW cm-2) irradiation (slow motion × 0.02) (MP4).

File Name: Supplementary Movie 3
Description: Bending simulation I of 1β crystal III based on measured surface temperature; 390 Hz nonresonated natural vibration with large photothermally driven bending upon UV laser irradiation for 100 ms and then turning off the UV light (slow motion × 0.02) (MP4).

File Name: Supplementary Movie 4
Description: Bending simulation I of 1β crystal III based on measured surface temperature; 390 Hz resonated natural vibration upon 390 Hz pulsed (1.282 ms on, 1.282 ms off) UV laser (375 nm, 1,456 mW cm-2) irradiation (slow motion × 0.02) (MP4).

File Name: Supplementary Movie 5
Description: Bending simulation II of 1β crystal III based on irradiated light energy; 390 Hz non-resonated natural vibration with large photothermally driven bending upon UV laser irradiation for 100 ms and then turning off the UV light (slow motion × 0.02) (MP4).

File Name: Supplementary Movie 6
Description: Bending simulation II of 1β crystal III based on irradiated light energy; 390 Hz resonated natural vibration upon 390 Hz pulsed (1.282 ms on, 1.282 ms off) UV laser (375 nm, 1,456 mW cm-2) irradiation (slow motion × 0.02) (MP4).
